# Let-7f-5p regulates TGFBR1 in glucocorticoid-inhibited osteoblast differentiation and ameliorates glucocorticoid-induced bone loss

**DOI:** 10.7150/ijbs.33490

**Published:** 2019-08-19

**Authors:** Geng-Yang Shen, Hui Ren, Qi Shang, Wen-Hua Zhao, Zhi-Da Zhang, Xiang Yu, Jin-Jing Huang, Jing-Jing Tang, Zhi-Dong Yang, De Liang, Xiao-Bing Jiang

**Affiliations:** 1 Guangzhou University of Chinese Medicine, Guangzhou 510405, China;; 2 Department of Spinal Surgery, The First Affiliated Hospital of Guangzhou University of Chinese Medicine, Guangzhou 510405, China;; 3 Lingnan Medical Research Center of Guangzhou University of Chinese Medicine, Guangzhou 510405, China;

**Keywords:** Let-7f-5p, osteoblast differentiation, bone formation, glucocorticoid-induced bone loss, TGFBR1, GR

## Abstract

Previous studies indicated that let-7 enhances osteogenesis and bone formation of human adipose-derived mesenchymal stem cells (MSCs). We also have confirmed that let-7f-5p expression was upregulated during osteoblast differentiation in rat bone marrow-derived MSCs (BMSCs) and was downregulated in the vertebrae of patients with glucocorticoid (GC)-induced osteoporosis (GIOP). The study was performed to determine the role of let-7f-5p in GC-inhibited osteogenic differentiation of murine BMSCs *in vitro* and in GIOP *in vivo*. Here, we report that dexamethasone (Dex) inhibited osteogenic differentiation of BMSCs and let-7f-5p expression, while increasing the expression of transforming growth factor beta receptor 1 (TGFBR1), a direct target of let-7f-5p during osteoblast differentiation under Dex conditions. In addition, let-7f-5p promoted osteogenic differentiation of BMSCs, as indicated by the promotion of alkaline phosphatase (ALP) staining and activity, Von Kossa staining, and osteogenic marker expression (*Runx2*,*Osx*, *Alp*, and *Ocn*), but decreased TGFBR1 expression in the presence of Dex. However, overexpression of TGFBR1 reversed the upregulation of let-7f-5p during Dex-treated osteoblast differentiation. Knockdown of TGFBR1 reversed the effect of let-7f-5p downregulation during Dex-treated osteogenic differentiation of BMSCs. We also found that glucocorticoid receptor (GR) mediated transcriptional silencing of let-7f-5p and its knockdown enhanced Dex-inhibited osteogenic differentiation. Further, when injected *in vivo*, agomiR-let-7f-5p significantly reversed bone loss induced by Dex, as well as increased osteogenic marker expression (*Runx2*, *Osx*, *Alp*, and *Ocn*) and decreased TGFBR1 expression in bone extracts. These findings indicated that the regulatory axis of GR/let-7f-5p/TGFBR1 may be important for Dex-inhibited osteoblast differentiation and that let-7f-5p may be a useful therapeutic target for GIOP.

## Introduction

Glucocorticoids (GCs) act as immunosuppressive agents and are widely prescribed for a range of inflammatory diseases, such as rheumatic diseases, as well as organ-transplant recipients. However, they are associated with well-established deleterious effects on bone that can result in GC-induced osteoporosis (GIOP), which is the most common cause of secondary osteoporosis. It was reported previously that almost 30-50% of adults will experience an osteoporotic fracture after long-term GC therapy, and furthermore, accelerated bone loss and increased fracture risk are evident soon after initiation of GC treatment 1. Mechanistically, reduced bone formation is regarded as a crucial process of GIOP and many studies have determined that the glucocorticoid receptor (GR), DKK1, sclerostin, AP‑1, PPARγ2, Runx2, and other critical molecules play key roles in this process 2-5. Additionally, this is the main difference between GIOP and postmenopausal osteoporosis (PMOP), the latter of which is characterized by increased bone turnover 6, 7. Although the 2017 American College of Rheumatology (ACR) guidelines 8 outlined how patients receiving GC therapy, should be managed, providing effective treatment for GIOP remains a challenge, due to the side-effects induced by existing recommended drugs, such as bisphosphonates and teriparatide 9. Therefore, it is necessary to investigate the cellular and molecular mechanisms by which GCs inhibit osteoblast differentiation and bone formation to understand the pathomechanism of GIOP and identify novel therapeutic targets.

MicroRNAs (miRNAs) are a class of small non-coding RNAs that regulate degradation or translational inhibition of transcripts by binding to complementary sites of target messenger RNAs 10-12. Emerging evidence suggest that miRNAs are involved in regulating bone homeostasis and osteoporosis 13, 14. In addition, there have been several studies regarding miRNA-mediated osteogenesis under GC treatment. Hypercortisolism in Cushing's disease patients inhibits osteoblastogenesis and osteoblast function by controlling the expression of miRNAs, including miR-125b-5p, miR-218-5p, miR-34a-5p, miR-188-3p, miR-199a-5p, which are known to suppress osteoblastogenesis 15. Another study indicated that miR-29a protected against GC-induced bone loss in rats and alleviated the inhibitory effects of GC on osteoblast differentiation and mineral acquisition by reducing GC-induced disturbance of Wnt and Dkk-1 actions 16. Also, GCs reduced miR-29a expression via HDAC4 activity and then reduced acetylation of histone 3 at lysine 9 in the miR-29a promoter 17. MiR-34a was reported to inhibit the effects of dexamethasone (Dex) on osteogenesis of mouse mesenchymal stem cells (MSCs) and angiogenesis, suggesting that it may be a useful therapeutic target for GC-induced osteonecrosis of the femoral head 18. However, the whole spectrum of miRNA-modulating osteoblast differentiation and bone formation under conditions of treatment with GCs *in vitro* and *in vivo* remains to be not fully elucidated.

In our previous study, high-throughput miRNA-sequencing analysis indicated that let-7f-5p miRNA, a subtype of let-7 family, was significantly downregulated in vertebrae of GIOP patients 19. Furthermore, we and other groups demonstrated that let-7 was a positive regulator in osteogenesis and bone formation of rat bone marrow-derived MSCs (BMSCs) 20 and human adipose-derived MSCs 21. In addition, let-7 has been shown to promote ectopic bone formation of human adipose-derived MSCs *in vivo* 21. Meanwhile, let-7 miRNAs acted as posttranscriptional regulators in blastodermal cells and T cells by repressing transforming growth factor beta receptor 1 (TGFBR1) 22, 23, a serine/threonine kinase receptor, to transform TGF-β signaling 24-26. Moreover, TGF-β and TGFBR1 promote osteoclast formation and inhibits osteoblast differentiation 27-33. However, the precise functions and underlying molecular mechanisms of action of let-7f-5p in GC-inhibited osteogenic differentiation of murine BMSCs and GC-induced bone loss in mice remain unclear. This study was performed to investigate the role of let-7f-5p in GC-inhibited osteoblast differentiation and the therapeutic potential of targeting let-7f-5p to treat GIOP. Using *in vitro* and *in vivo* approaches, we showed that let-7f-5p rescued Dex-inhibited osteogenic differentiation of murine BMSCs and Dex-induced bone loss by targeting TGFBR1, a negative regulator of osteogenesis. These observations suggested that targeting let-7f-5p may provide novel therapeutic options for the prevention and treatment of GIOP.

## Materials and Methods

### Cell isolation and culture

The cells were cultured in a modified essential medium (α-MEM) containing 10% foetal bovine serum (FBS), 100 units/ml penicillin, and 100 mg/ml streptomycin (Gibco). BMSCs derived from mice were harvested and cultured as follows. 8-week-old mice were purchased in the Experimental Animal Center of Guangzhou University of Chinese Medicine (Guangzhou, China). After euthanasia, we removed the long bones (tibiae and femurs) aseptically and flushed out the bone marrow with α-MEM supplemented with 20% FBS and 1% Penicillin-Streptomycin. The cells were filtered with a 40‐μm cell strainer and cultured in 35‐mm dishes at a density of 4×10^4^/cm^2^ at 37°C in 5% CO_2_ for 4 days. We collected the cells in fraction 2 to 6 for pre‐osteoblasts cultures and osteogenic differentiation experiments.

### Osteogenic differentiation and treatment

For the osteogenic differentiation, unattached cells were removed and replaced with osteogenic induction medium (10% FBS in a‐MEM containing 25mg/mL Vit C and 5mM β‐Glycerophosphate) with or without Dex (0.1 μM) in 12-well cell culture plates as previously described 18, which were then replaced with the osteogenic induction medium and Dex every 2-3 days. After 5 to 7 days in osteogenic induction medium, the cells were used for alkaline phosphatase (ALP) staining and ALP activity assay. Mineralization typically occurs after 10 to 14 days in culture, and the cells were stained with the Von Kossa method for measurement of mineralized nodule formation.

### ALP staining, ALP activity, and mineralization assay

After osteogenic induction for 7 days, we fixed the cells with 4% formaldehyde (Sigma, Shanghai, China) for 15 min at room temperature, and incubated them with ALP substrate, BCIP/NBT (Thermo Scientific Waltham, MA). To test the ALP activity, we lysed the cells by using a radio immunoprecipitation assay (RIPA, Beyotime, Shanghai, China) and then determined the ALP activity through performing an ALP Activity Assay (Beyotime). For measurement of mineralized nodule formation, cells were fixed with 4% formaldehyde and washed with PBS for 3 times. Then, we incubated the cells with a 5% silver nitrate solution and exposed them under the light for 30 min. Lastly, we used a 5% sodium thiosulfate to remove the nonspecific staining for 5 min.

### Prediction of let-7f-5p target genes

Let-7f-5p target genes and the binding sites were predicted by using diverse bioinformatic platforms, such as TargetScan 7.2 (http://targetscan.org), miRBase, miRDB, miRanda, etc.

### MiRNAs and reporter vectors construction

Let-7f-5p, mutation constructs, and reporter gene construction were performed according to previous studies 20. Briefly, we used genomic DNA from mouse as the template and the genomic fragments of let-7f-5p precursors were amplified by reverse transcription PCR (RT-PCR). Next, we cloned the amplified products into the restriction sites (NotI and XhoI) of pLAS2-RFP vector. Then BMSCs was virally infected with the modified vector and let-7f-5p expression was detected by quantitative RT-PCR (qRT-PCR). Additionally, we cloned the let-7f-5p's binding site in TGFRB1 and the whole TGFRB1 3′UTR sequence into the restriction sites (PmeI and XhoI) of pmirGLO luciferase vector. Also, a pair of primers with mutant sequence were used to generate the mutation constructs of TGFRB1 3′UTR.

### Transfection of let-7f-5p mimics and antagomiR

MiRIDIAN miRNA mimics were used to design the let-7f-5p overexpression. Anti-let-7f-5p miRNA inhibitors (AntagomiR) were purchased from Dharmacon (Denver, CO). BMSCs were transfected for 24 h with let-7f-5p mimics (100 nM), let-7f-5p antagomiR (100 nM) or miR-NC (negative control, 100 nM) through Lipofectamine 2000 reagent. Then the cells were used for the following experiments.

### RT-PCR and qRT-PCR

RT-PCR and qRT-PCR were performed as described elsewhere 20. Firstly, total RNA was extracted from BMSCs or bone tissues with Trizol (Sigma). Then, to obtain cDNA, we diluted 1 μg of RNA with 10 ml of nuclease-free water. Then we added into 1 μl of 50 mM hexamer primers. Next, the denatured process of the solution was performed with respective temperature and time point sequentially (65°C, 5 min; and 4°C, 60 min). Lastly, the solution was incubated with at 25°C for 10 min, 45°C for 60 min and and 75°C for 5 min. The primers for qRT-PCR were listed at **Table S1**. Additionally, for miRNA qRT-PCR assay, total RNA was isolated and the small RNA fractions were enriched using mirVana miRNA Isolation Kit. Next, the cDNA was prepared with the TaqMan miRNA Reverse Transcription Kit and RT-PCR was performed. Lastly, let-7f-5p expression level was quantified by TaqMan U6 snRNA assay.

### Western blot analysis

To investigate protein expression levels in cells and bones, western blot was performed. The protein samples were subjected with equal protein concentration to SDS-polyacrylamide gel electrophoresis (SDS-PAGE) and transferred to PVDF membranes (Milipore, Darmstadt, Germany). Next, membranes were blotted with 5% skim milk-PBS-Tween 20 for 1 h at room temperature and incubated with primary antibodies overnight at 4°C. Rabbit anti-TGFBR1 (1:1,000, sc-101574, Santa Cruz) and rabbit anti-GR (1:1000, sc-56851, Santa Cruz) were used. After that, the blots were washed with PBS-Tween 20 (PBST) and incubated with horseradish peroxidase (HRP)-conjugated secondary antibodies (1:3000) for 1 h at room temperature. Lastly, the blots were washed again and incubated with enhanced chemilumescent (ECL) substrates (Bio-rad) for 1 min and the Image J software was applied to analysing the blots.

### Gene overexpression and knockdown assay

For the overexpression of TGFBR1, TGFBR1 CDS region was amplified from mouse cDNA and cloned into pCS2+ plasmid vector which included the CMV promoter. For the silence of TGFBR1 and GR, small interfere RNA (siRNA), composed of several target-specific 19-25-nucleotide siRNAs, was used (Santa Cruz, Dallas, Texas) and BMSCs were transfected with Lipofectamine RNAimax (Invitrogen) according to the manufacturer's instruction. The overexpression and knockdown efficiency of TGFBR1 and GR were examined using qRT-PCR.

### Dual-luciferase reporter assay

Luciferase activity of the reporter constructs were evaluated by performing the dual-luciferase reporter assay (Promega, USA). In brief, BMSCs (80% confluence) were seeded in the 6-well plates and were induced the differentiation for 24 h. Then the cells were cotransfected with 100 nM of let-7f-5p mimics, 1 μg of TGFBR1 3′UTR or the control, and miR-NC per well by using Lipofectamine 2000. After 24 h of transfection, cell extracts were prepared for measuring the luciferase activity through the Dual-Luciferase Reporter Assay System (Promega, Biotech Co., Ltd., China).

### ChIP assay

Chromatin immunoprecipitation (ChIP) assay was carried out in line with the manufacturer's instructions (Millipore, Darmstadt, Germany). After the intervention process, BMSCs were fixed through 1% paraformaldehyde and then lysed with SDS lysis buffer. Followed by which, the lysate was sonicated (16 rounds of 20 pulses with 2 minutes between rounds) and the protein-DNA mixture were immunoprecipitated by using IgG or anti-GR antibody at 4°C overnight. Then the products were collected after incubation on protein A + G-coated magnetic beads. The beads were washed and ChIP elution buffer was used to elute the bound chromatin. Proteinase K was applied to digest the protein for 4 h at 45°C. DNA purification kit was used to purify the DNA. The DNA fragments of the GR binding sites in the let-7f-5p promoter were designed and synthesized by RiboBio (Guangzhou, China). Next, qPCR was applied to test the GR binding site. The total chromatin was regarded as input and IgG was used as controls.

### GC-induced bone loss

For GIOP animals model, 8-week-old male C57BL/6 mice (n=6 per group) (Experimental Animal Center of Guangzhou University of Chinese Medicine, Guangzhou, China) were intraperitoneally administered with Dex (the First Affiliated Hospital of Guangzhou University of Chinese Medicine, Guangzhou, China) daily at a dose of 1 mg/kg body weight as described 34-36. Animals were housed in cages under pathogen-free conditions under a 12-hlight and 12-h dark cycle in the animal room of the First Affiliated Hospital of Guangzhou University of Chinese Medicine (approval no. SYXK [Yue] 2018-0001) under a 12-h/12-h light/dark cycle (lightson at 08:00) with free access to a standard rodent diet and water. After 8 weeks of treatment, vertebrae were dissected and used for micro-CT and histomorphometric analysis.

### Micro-CT analysis

Micro-CT examination was accomplished as described previously 37 through a Skyscan 1172 micro-CT imaging system (Skyscan, Kontich, Belgium) with a 12 mm spatial resolution (X-ray source 80 kV/100μA). Volume reconstruction was achieved via the built-in software NRecon 1.6 and CTAn 1.8, respectively. To analyze the bone remodeling, the volume of interest (VOI) was characterized as a cylindrical space wrapping the cancellous bone. Trabecular bone volume (BV/TV, %), trabecular number (Tb.N, /mm), trabecular thickness (Tb.Th, mm), and trabecular separation (Tb.Sp, mm) were calculated within the delimited VOI.

### Bone histomorphometry

For histological analysis, the mice were injected with 25 mg/kg calcein at 8 and 2 days before euthanasia. Preparation of undecalcified vertebrae samples and trabecular bone histomorphometry were executed in accordance with previous studies 38. Bone samples were fixed with 10% paraformaldehyde overnight and reserved in 70% ethanol at 4℃, and then were embedded with methylmethacrylate. Five-mm-thick pieces were processed through the microtome. Histomorphometric analysis of the L4 vertebrae mainly contained bone formation parameters bone formation rate (BFR/BS, μm^3^ μm^-2^ per day), mineral apposition rate (MAR, μm per day), mineralizing surface (MS/BS, %), and osteoblast number (N.Ob/B.Pm, /mm). All above parameters were measured and expressed as stated in ASBMR nomenclature committee 39.

### ELISA

The concentration of serum procollagen type 1 N-terminal propeptide (P1NP) and osteocalcin (OCN) were measured using commercial enzyme-linked immunosorbent assay (ELISA) kits from IDS (Fountain Hills, AZ). Blood was collected by puncturing the mice cheekpouch. And the mice were fasted for 4 h before collecting blood.

### Statistical analysis

We conducted three independent experiments on all experiments. All determined parameters were expressed as mean ± standard deviation (s.d.). The significant statistical differences were appraised using two‐tailed unpaired Independent-Sample T test for comparisons between two groups. One‐way Analysis of Variance (ANOVA) plus the Tukey's test were used for comparisons among three or more groups. *P* value < 0.05 was recognized to have statistically significant differences.

## Results

### Dex inhibited let-7f-5p but promoted TGFBR1 expression during osteogenic differentiation of BMSCs and let-7f-5p modulated TGFBR1

We first explored the roles of Dex on proliferation and osteoblastic differentiation of BMSCs. The presence of Dex markedly reduced the proliferation and osteogenesis potential of BMSCs, which is evident in CCK-8 assay, alkaline phosphatase (ALP) staining, and Von Kossa staining (**Figure S1A-C**). Quantitative analysis confirmed the inhibition of ALP activity (**Figure S1D**), an early marker of osteogenesis. Furthermore, qRT-PCR analysis revealed significant reductions in the mRNA expression levels of the key osteogenic markers *Runx2*, *Osx* (Osterix), *Alp*, and *Ocn* (Osteocalcin) in BMSCs with Dex treatment in comparison to the controls (**Figure S1E**).

**Figure 1:**
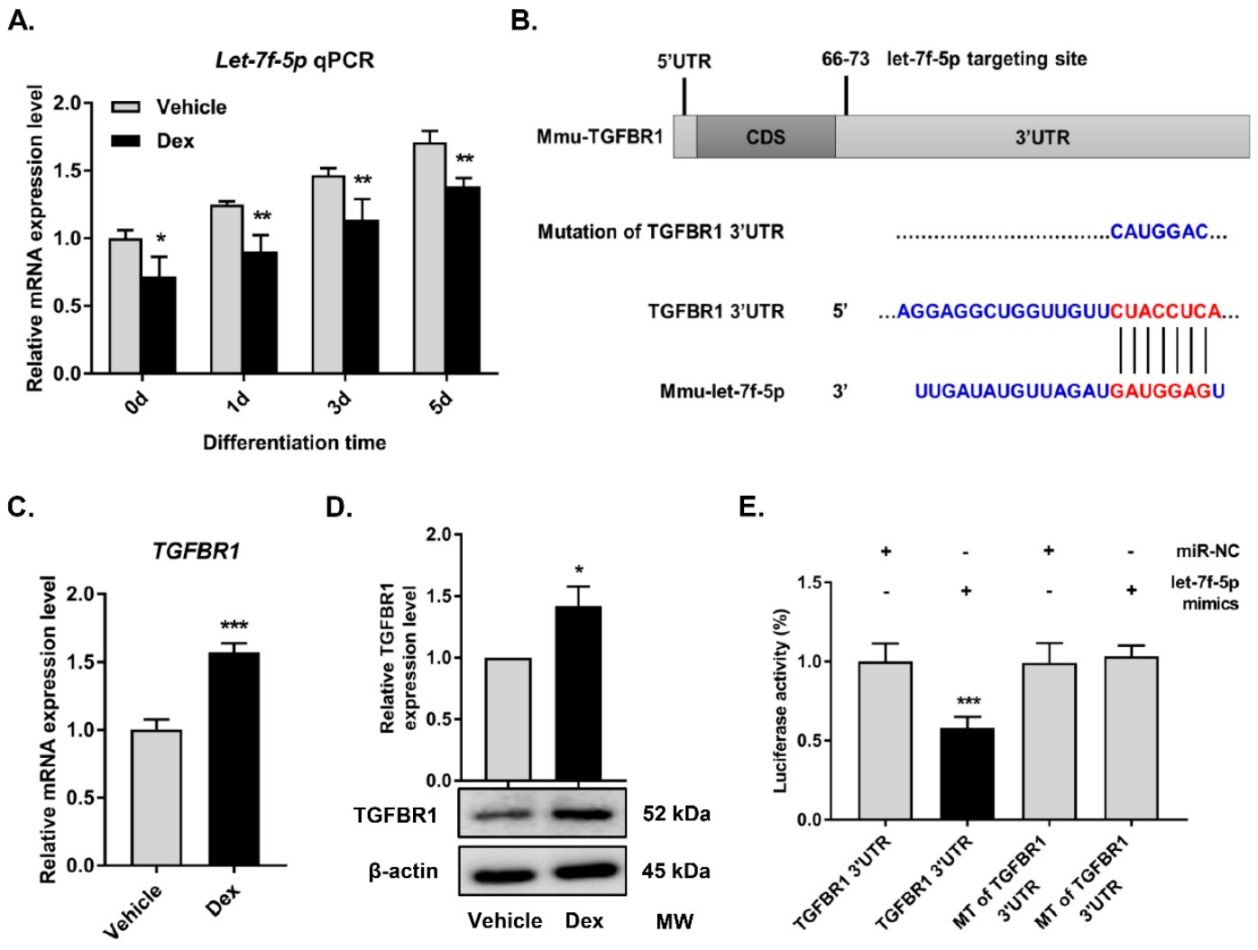
** Dexamethasone (Dex) inhibits let-7f-5p expression and TGFBR1 is a direct target for let-7f-5p during osteogenic differentiation of BMSCs under Dex conditions.** (A) Dex reduced *let-7f-5p* expression during differentiation (days 0, 1, 3, and 5) in BMSCs. Results are shown as mean ± s.d. of three independent experiments performed in triplicates, ^*^P < 0.05 and ^**^P < 0.01 by *t* test. (B) Base pairing comparison between mature let-7f-5p and WT or MT TGFBR1 3′UTR putative target site was shown according to the TargetScan database. (C) Increased mRNA expression of *TGFBR1* in Dex treated BMSCs. Results are shown as mean ± s.d. of three independent experiments performed in triplicates, ^***^P < 0.001 by *t* test. (D) Dex stimulated protein expression of TGFBR1 in BMSCs. Results are shown as mean ± s.d. of three independent experiments performed in triplicates, ^*^P < 0.05 by *t* test. (E) Luciferase reporter assay showed marked reduction of the 3′UTR of TGFBR1-WT by let-7f-5p mimics. Results are shown as mean ± s.d. of three independent experiments performed in triplicates, ^***^P < 0.001 by ANOVA with Tukey's *post hoc* test.

Our previous study using high-throughput miRNA-sequencing analysis revealed a marked reduction of let-7f-5p in vertebrae of GIOP patients and indicated that let-7f-5p acted as a positive modulator of osteoblast proliferation and differentiation *in vitro* 18-20. Here, we further examined whether Dex has a regulatory effect on osteogenic differentiation of BMSCs by targeting let-7f-5p *in vitro*. Quantitative RT-PCR assay showed that the expression levels of let-7f-5p during osteogenic differentiation of BMSCs were significantly reduced on days 1, 3, and 5 with Dex treatment (**Figure 1A**), suggesting that Dex could repress let-7f-5p expression during osteogenic differentiation of BMSCs.

MiRNAs function as regulators of multiple biological activities through suppression of target gene expression. With regard to the promotion of osteogenesis by let-7f-5p, we attempted to identify the candidate protein-coding genes targeted by let-7f-5p. Among the candidates predicted by the TargetScan bioinformatics platform (http://targetscan.org), TGFBR1 was found to be of interest (**Figure 1B**). The TGF-β signalling pathway has been reported to play a crucial role in bone homeostasis, inducing osteoclast formation and repressing osteoblast differentiation, and TGFBR1 acts as an important signalling factor. Further, we found that Dex increased TGFBR1 mRNA and protein expression levels in BMSCs (**Figure 1C-D**). Therefore, to determine whether let-7f-5p binds directly to 3′UTR of TGFBR1, we performed luciferase reporter assays by constructing a whole length of TGFBR1 3′UTR, which was recognized as the TGFBR1 wild-type (WT) 3′UTR by subcloning in a pmir-GLO dual-luciferase reporter. The subcloned site is 3′ end in firefly luciferase coding sequence. The TGFBR1 mutant (MT) 3′UTR was designed by mutating the binding sequence from CTACCTCA to CATGGAC (**Figure 1B**). Following let-7f-5p mimics transfection, the activity of WT was significantly repressed compared with that of MT (**Figure 1E**). All these data revealed the selectivity of let-7f-5p regulation to TGFBR1 3′UTR.

### Let-7f-5p negatively regulated TGFBR1 and promoted osteogenic differentiation of Dex-treated BMSCs

**Figure 2:**
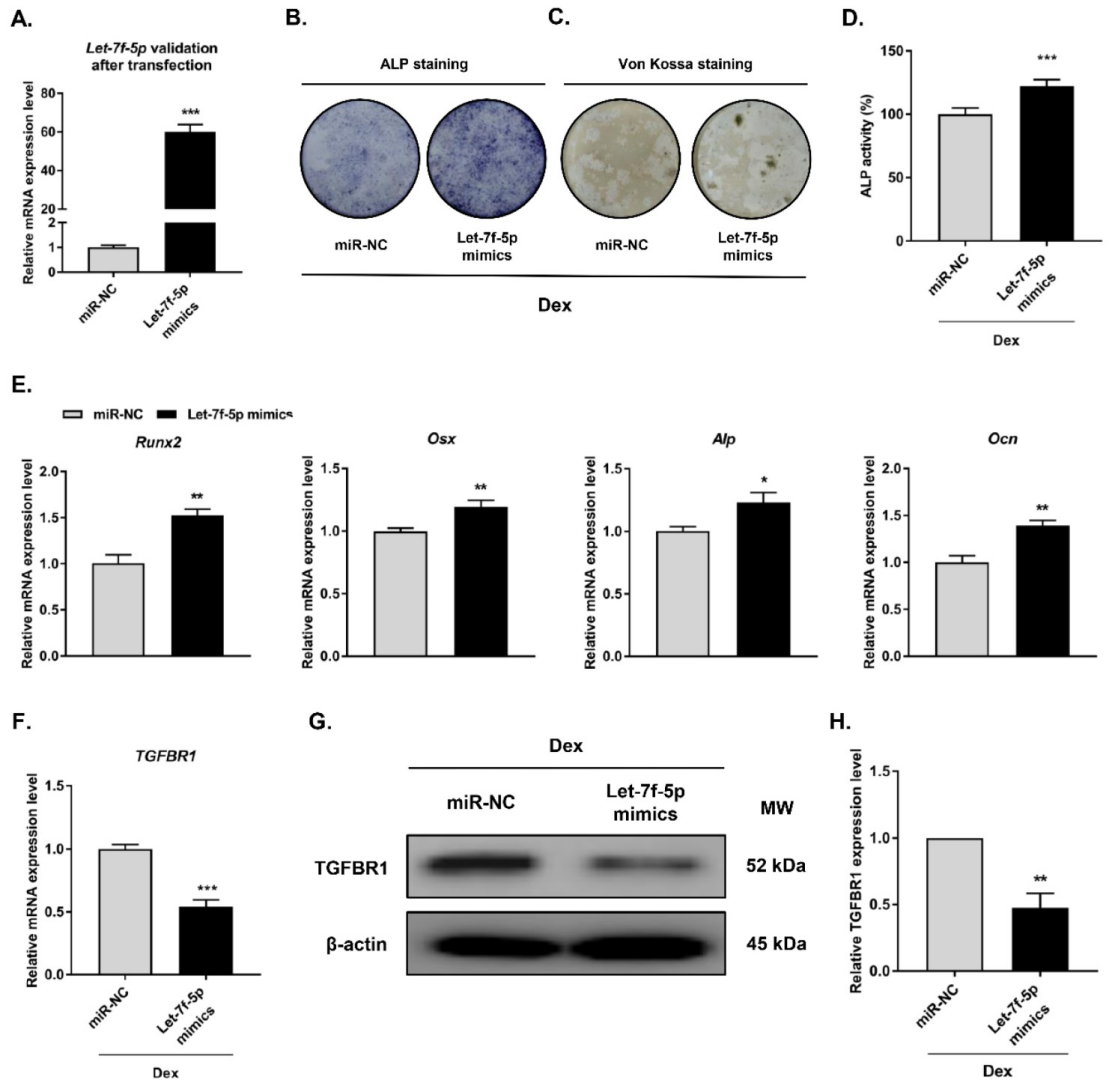
** Let-7f-5p promotes osteogenic differentiation of BMSCs by targeting TGFBR1 under Dex conditions.** (A) Increased let-7f-5p expression in BMSCs transfected with let-7f-5p mimics. Results are shown as mean ± s.d. of three independent experiments performed in triplicates, ^***^P < 0.001 by *t* test. (B-C) ALP staining and Von Kossa staining of BMSCs transfected with let-7f-5p mimics with Dex treatment. (D) Quantitative analysis of the ALP activity in BMSCs transfected with let-7f-5p mimics with Dex treatment. Results are shown as mean ± s.d. of three independent experiments performed in triplicates, ^***^P < 0.001 by *t* test. (E) Increased mRNA expression of *Runx2*, *Osx*, *Alp*, and *Ocn* in BMSCs transfected with let-7f-5p mimics with Dex treatment. Results are shown as mean ± s.d. of three independent experiments performed in triplicates, ^*^P < 0.05 and ^**^P < 0.01 by *t* test. (F-H) let-7f-5p inhibited mRNA expression and protein expression of TGFBR1 in BMSCs under Dex conditions. Results are shown as mean ± s.d. of three independent experiments performed in triplicates, ^**^P < 0.01 and ^***^P < 0.001 by *t* test.

To investigate the regulatory effect of let-7f-5p in Dex-inhibited osteogenic differentiation of BMSCs, we carried out gain-of-function experiments through transfecting let-7f-5p mimics into BMSCs prior to Dex-inhibited osteogenic differentiation of BMSCs (**Figure 2A**). Osteogenic differentiation and mineralisation activity of BMSCs as demonstrated by ALP staining (**Figure 2B**), Von Kossa staining (**Figure 2C**), and quantitative ALP activity (**Figure 2D**), were promoted by let-7f-5p mimics. And the marker genes (*Runx2*, *Osx*, *Alp*, and *Ocn*) of osteoblast differentiation exhibited increased expression after transfection of let-7f-5p mimics (**Figure 2E**). Further, overexpression of let-7f-5p markedly decreased TGFBR1 expression at the mRNA and protein levels (**Figure 2F-H**). Our data indicated that let-7f-5p restores Dex-inhibited osteogenic differentiation of BMSCs by targeting the 3′-UTR of TGFBR1. In addition, let-7f-5p was also shown to downregulate TGFBR1 expression and promoted promote the development of osteogenic differentiation phenotypes of BMSCs without Dex treatment (**Figure S2**).

### Overexpression of TGFBR1 reversed the effects of let-7f-5p upregulation on osteogenic differentiation of Dex-treated BMSCs

**Figure 3:**
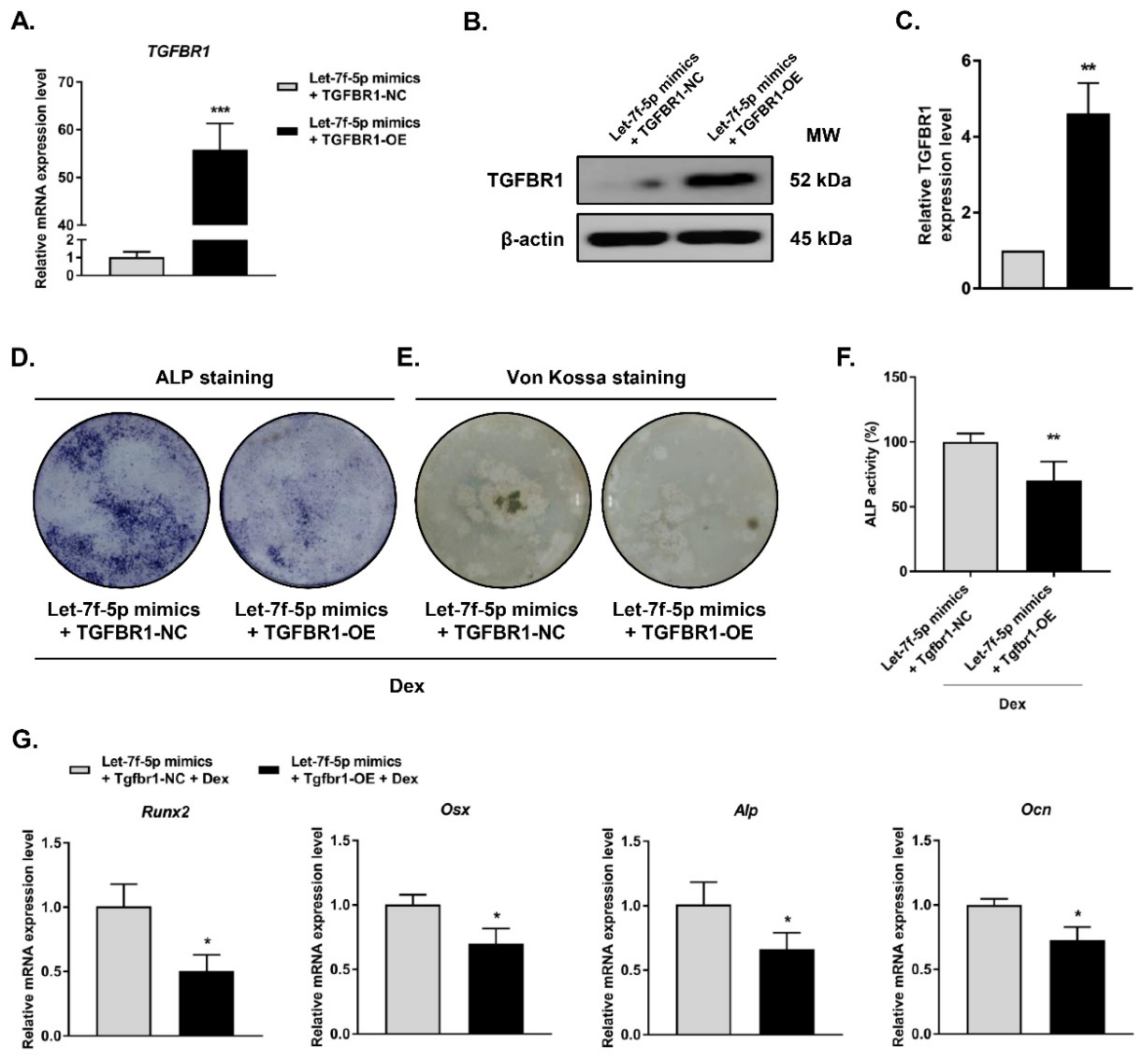
**Overexpression of TGFBR1 reverses the effect of the upregulation of let-7f-5p on osteogenic differentiation of BMSCs under Dex conditions.** (A-C) Increased mRNA and protein expression of TGFBR1 in BMSCs transfected with let-7f-5p mimics with overexpression of TGFBR1. Results are shown as mean ± s.d. of three independent experiments performed in triplicates, ^**^P < 0.01 and ^***^P < 0.001 by *t* test. (D-E) ALP staining and Von Kossa staining of BMSCs transfected with let-7f-5p mimics with overexpression of TGFBR1 under Dex conditions. (F) Quantitative analysis of the ALP activity in BMSCs transfected with let-7f-5p mimics with overexpression of TGFBR1 under Dex conditions. Results are shown as mean ± s.d. of three independent experiments performed in triplicates, ^**^P < 0.01 by *t* test. (G) Inhibited mRNA expression of *Runx2*, *Osx*, *Alp*, and *Ocn* in BMSCs transfected with let-7f-5p mimics with overexpression of TGFBR1 under Dex conditions. Results are shown as mean ± s.d. of three independent experiments performed in triplicates, ^*^P < 0.05 by *t* test.

To examine whether TGFBR1 gain-of-function can reverse the promotion of Dex-treated osteogenic differentiation by let-7f-5p mimics, we co-transfected the TGFBR1 overexpression plasmid and let-7f-5p mimics into BMSCs. TGFBR1 expression was markedly upregulated in comparison to the controls (**Figure 3A-C**). We next evaluated the effects of overexpressing TGFBR1 together with let-7f-5p mimics on osteogenic differentiation and mineralisation activity of Dex-treated BMSCs. Compared with the control group, reduced levels of ALP staining, fewer mineralization nodules, and decreased ALP activity were observed in the group with TGFBR1 overexpression and let-7f-5p mimics (**Figure 3D-F**). In addition, the upregulation of osteospecific genes including *Runx2*, *Osx*, *Alp*, and *Ocn* messenger RNA induced by let-7f-5p mimics in BMSCs was also reversed by a combination of TGFBR1 overexpression and let-7f-5p mimics in BMSCs (**Figure 3G**).

### Knockdown of TGFBR1 reversed the effects of downregulation of let-7f-5p on osteogenic differentiation of Dex-treated BMSCs

**Figure 4:**
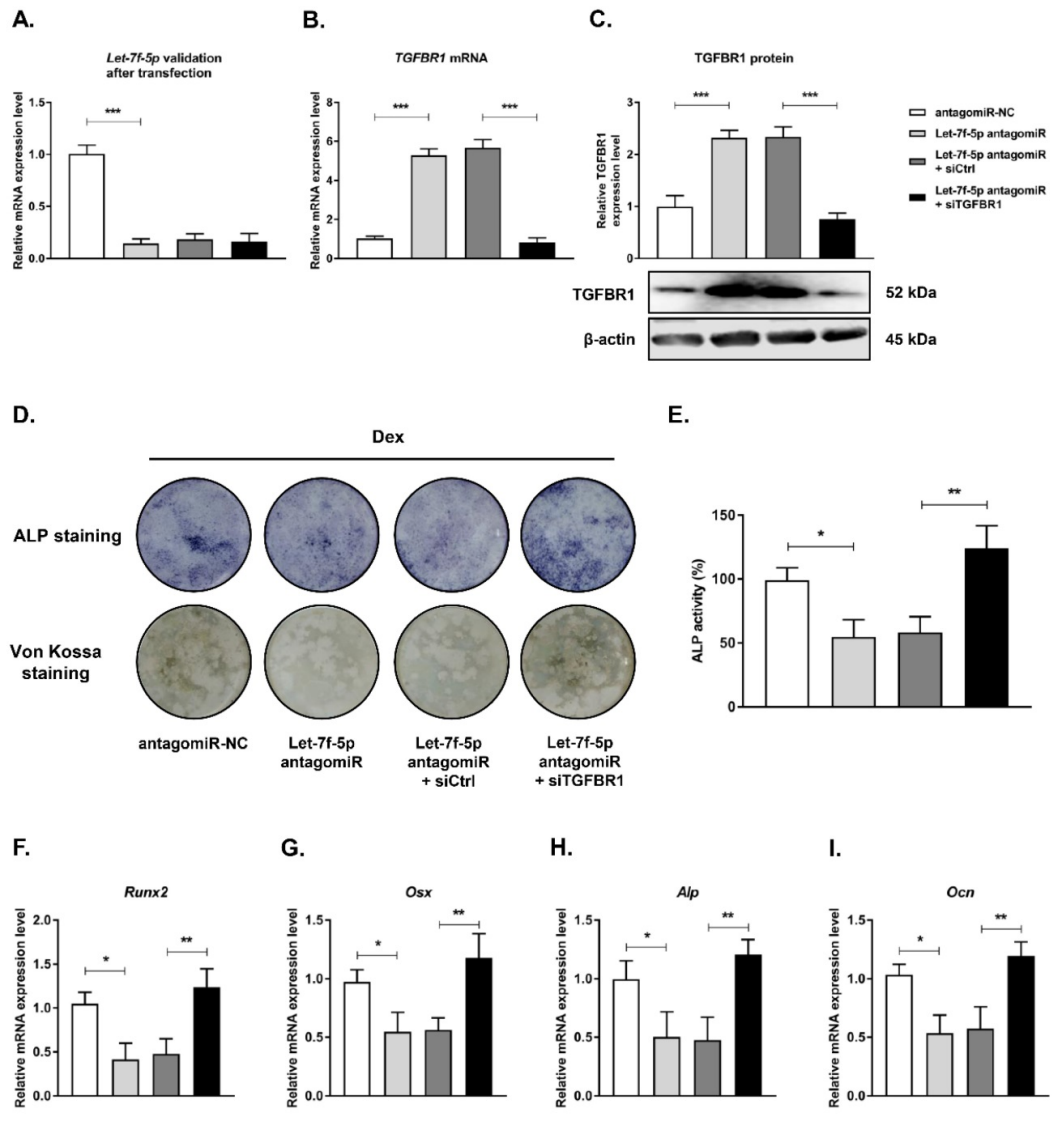
** Knockdown of TGFBR1 reverses the effect of the downregulation of let-7f-5p on osteogenic differentiation of BMSCs under Dex conditions.** (A) mRNA expression of let-7f-5p in BMSCs. Results are shown as mean ± s.d. of three independent experiments performed in triplicates, ^***^P < 0.001 by ANOVA with Tukey's *post hoc* test. (B-C) mRNA and protein expression of TGFBR1 in BMSCs. Results are shown as mean ± s.d. of three independent experiments performed in triplicates, ^***^P < 0.001 by ANOVA with Tukey's *post hoc* test. (D) ALP staining and Von Kossa staining of BMSCs transfected with let-7f-5p antagomiR with siTGFBR1 under Dex conditions. (E) Quantitative analysis of the ALP activity in BMSCs transfected with let-7f-5p antagomiR with siTGFBR1 under Dex conditions. Results are shown as mean ± s.d. of three independent experiments performed in triplicates, ^*^P < 0.05 and ^**^P < 0.01 by ANOVA with Tukey's *post hoc* test. (F-I) mRNA expression of *Runx2*, *Osx*, *Alp*, and *Ocn* in BMSCs transfected with let-7f-5p antagomiR with siTGFBR1 under Dex conditions. Results are shown as mean ± s.d. of three independent experiments performed in triplicates, ^*^P < 0.05 and ^**^P < 0.01 by ANOVA with Tukey's *post hoc* test.

Next, we examined the role of TGFBR1 loss-of-function in reversing the inhibition of osteogenic differentiation of Dex-treated BMSCs with let-7f-5p antagomiR by co-transfecting TGFBR1 siRNA and let-7f-5p antagomirs into BMSCs. Let-7f-5p and TGFBR1 expression levels were verified after transfection (**Figure 4A-C**). The effects of TGFBR1 knockdown combined with let-7f-5p antagomiR on osteogenic differentiation and mineralisation activity of Dex-treated BMSCs were examined. The let-7f-5p antagomiR significantly inhibited ALP positive staining, mineralisation nodule formation, and ALP activity. The group treated with let-7f-5p antagomir and TGFBR1 siRNA exhibited increased ALP staining, more mineralisation nodules, and increased ALP activity relative to the controls (**Figure 4D-E**). In addition, expression trends of osteospecific genes (*Runx2, Osx, Alp, and Ocn*) were consistent with the results of ALP staining and the mineralisation assay (**Figure 4F-I**). These results indicated that TGFBR1 acted as a negative regulator for the promotion of osteogenic differentiation of Dex-treated BMSCs by let-7f-5p.

### GR suppressed let-7f-5p expression under Dex treatment

**Figure 5:**
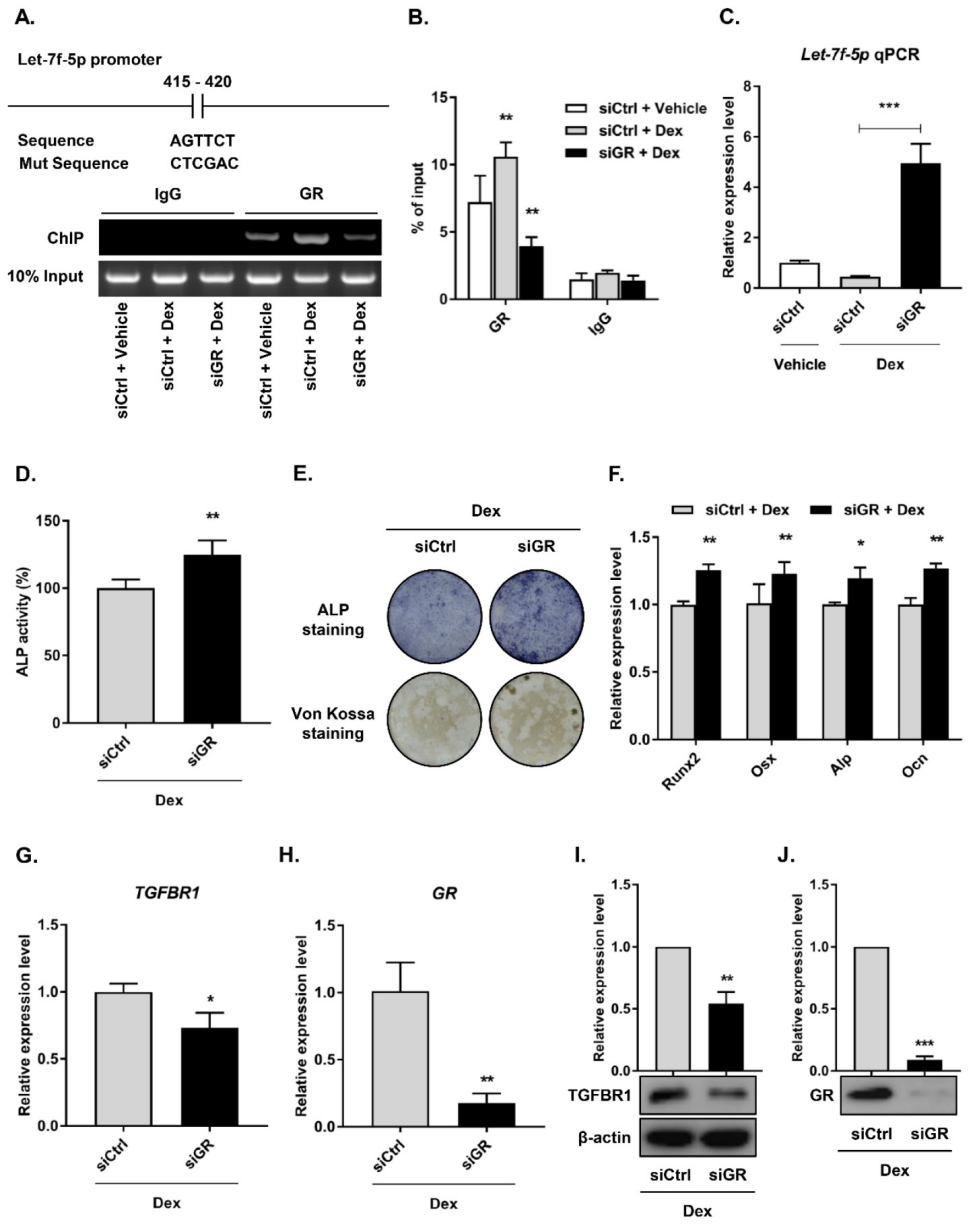
** GR mediates transcriptional silencing of let-7f-5p during Dex-inhibited osteogenic differentiation of BMSCs.** (A) The predicted GR binding site in let-7f-5p promoter and the mutant versions are shown. (B) ChIP assay revealed the significant reduction in binding of GR with the let-7f-5p promoter in BMSCs transfected with siGR under Dex conditions. Results are shown as mean ± s.d. of three independent experiments performed in triplicates, ^**^P < 0.01 by ANOVA with Tukey's *post hoc* test. (C) siGR promoted the mRNA expression of let-7f-5p under Dex conditions. Results are shown as mean ± s.d. of three independent experiments performed in triplicates, ^***^P < 0.001 by ANOVA with Tukey's *post hoc* test. (D) Quantitative analysis of the ALP activity in BMSCs transfected with siGR under Dex conditions. Results are shown as mean ± s.d. of three independent experiments performed in triplicates, ^**^P < 0.01 by *t* test. (E) ALP staining and Von Kossa staining of BMSCs transfected with siGR under Dex conditions. (F) Increased messenger RNA expression of *Runx2*, *Osx*, *Alp*, and *Ocn* in BMSCs transfected with siGR under Dex conditions. Results are shown as mean ± s.d. of three independent experiments performed in triplicates, ^*^P < 0.05 and ^**^P < 0.01 by *t* test. (G-J) Reduced mRNA and protein expression of TGFBR1 and GR in BMSCs transfected with siGR under Dex conditions. Results are shown as mean ± s.d. of three independent experiments performed in triplicates, ^*^P < 0.05, ^**^P < 0.01, and ^***^P < 0.001 by *t* test.

Most roles of GC are modulated by the GR, an extensively expressed nuclear receptor. GCs suppress bone formation by attenuating osteoblast differentiation via monomeric GR 2. To determine whether the GR directly regulated let-7f-5p expression, we performed loss-of-function experiments using siRNAs to knock down GR expression in the BMSCs (siGR), and then confirmed the knockdown effect (**Figure 5H and J**) . The control group was termed “siCtrl”. Next, we verified transcriptional regulation through ChIP experiment using an anti-GR antibody on the let-7f-5p promoter. The results indicated that GR binding was significantly reduced at the let-7f-5p promoter in the siGR cells compared to siCtrl cells (**Figure 5A**). In addition, GR knockdown markedly increased the level of cellular let-7f-5p expression (**Figure 5B**).

To elucidate the effect of GR-mediated let-7f-5p in Dex-inhibited osteogenic differentiation of BMSCs, we performed downstream functional analysis, including assays of osteogenic differentation, mineralisation activity, osteogenic differentation marker expression level, and TGFBR1 expression level, by transfecting siGR into BMSCs. Levels of ALP activity (**Figure 5C**), ALP staining (**Figure 5D**), Von Kossa staining (**Figure 5E**), and expression of *Runx2*, *Osx*, *Alp*, and *Ocn* mRNA increased in the GR-knockdown cells (**Figure 5F**). Further, GR loss-of-function markedly inhibited the mRNA and protein expression levels of TGFBR1 (**Figure 5G-I**). Taken together, these observations suggest that GR may suppress let-7f-5p expression at the transcriptional level during Dex-inhibited osteogenic differentiation of BMSCs.

### Let-7f-5p reversed Dex-induced bone loss in mice

**Figure 6:**
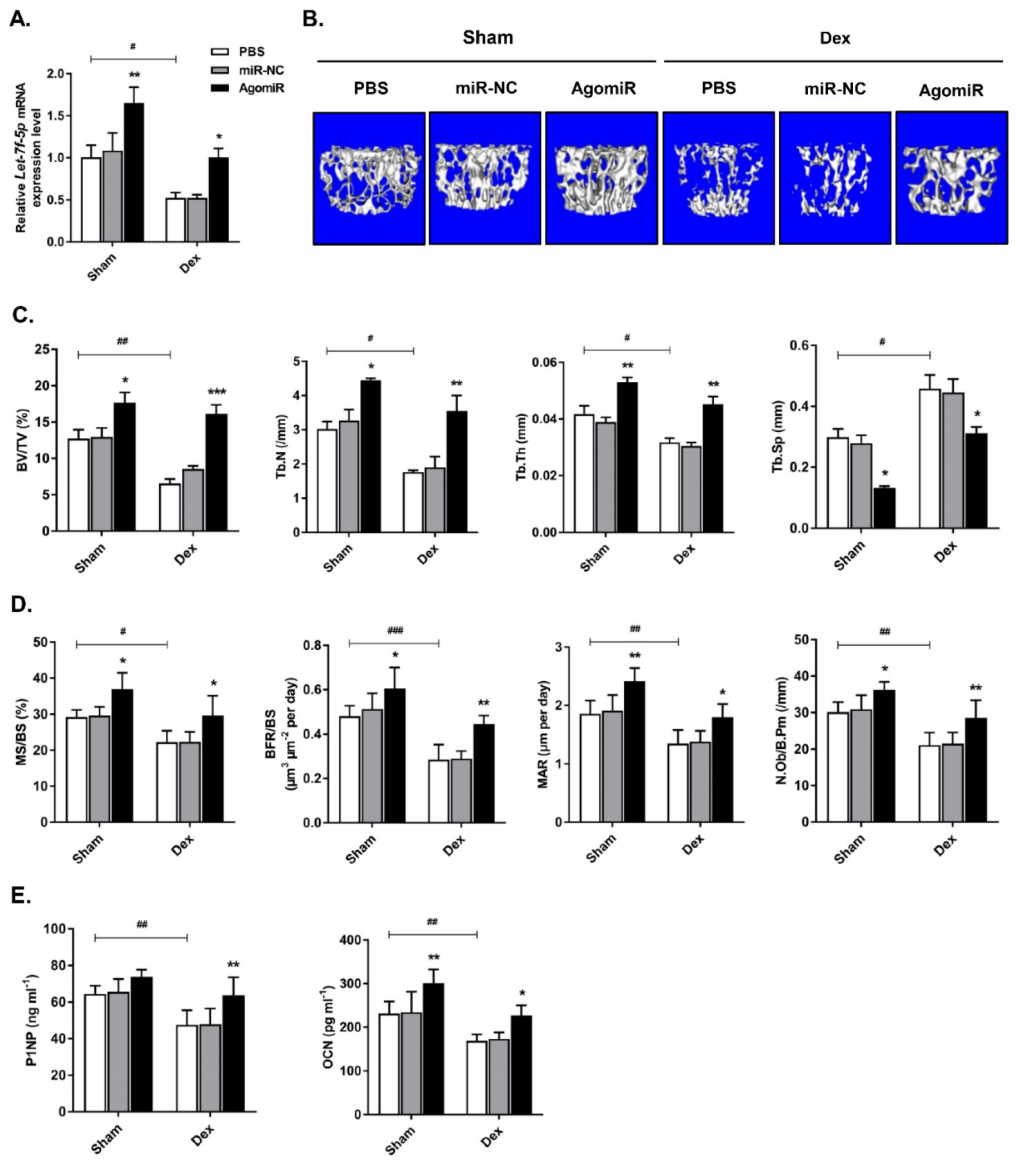
** Let-7f-5p reverses Dex-induced bone loss in mice.** (A) Increased let-7f-5p expression in bone following injection of let-7f-5p (agomir) using Invivofectamine 2.0 reagent into 6-week-old female C57 mice. Results are shown as mean ± s.d., n=6, ^*^P < 0.05, ^**^P < 0.01, and ^#^*P* < 0.05 by ANOVA with Tukey's *post hoc* test. (B) micro-CT scanning of lumbar 4 vertebrae. (C-D) Histomorphometric analysis of the trabecular bone in vertebrae. Results are shown as mean ± s.d., n=6, ^*^P < 0.05, ^**^P < 0.01, ^***^P < 0.001,^ #^*P* < 0.05, ^##^*P* < 0.01, and ^###^*P* < 0.001 by ANOVA with Tukey's *post hoc* test. (E) The serum levels of P1NP and OCN. Results are shown as mean ± s.d., n=6, ^*^P < 0.05, ^**^P < 0.01, and^ ##^*P* < 0.01 by ANOVA with Tukey's *post hoc* test.

**Figure 7:**
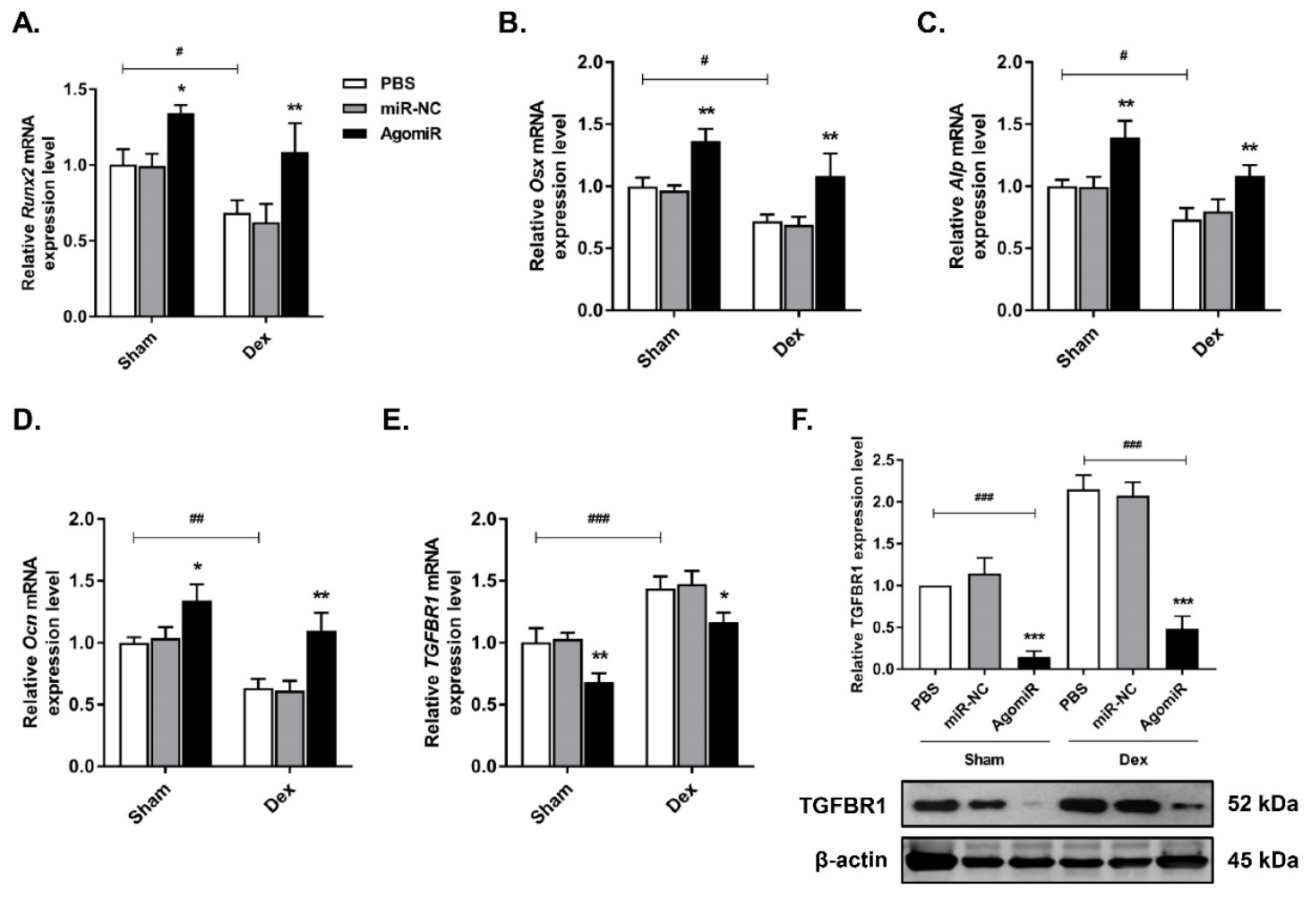
** Osteogenic markers and TGFBR1 expression are affected by the presence of let-7f-5p in mice.** (A-D) Increased *Runx2*, *Osx*, *Alp*, and *Ocn* expression in bone extracts following injection of let-7f-5p (agomir) from mice. Results are shown as mean ± s.d., n=6, ^*^P < 0.05, ^**^P < 0.01, ^#^*P* < 0.05, ^##^*P* < 0.01, and ^###^*P* < 0.001 by ANOVA with Tukey's *post hoc* test. (E-F) Reduced mRNA and protein expression of TGFBR1 in bone extracts following injection of let-7f-5p (agomir) from mice. Results are shown as mean ± s.d., n=6, ^*^P < 0.05, ^**^P < 0.01, and ^###^*P* < 0.001 by ANOVA with Tukey's *post hoc* test.

To investigate the anabolic function of let-7f-5p *in vivo*, we injected Dex-treated or sham-operated control mice with 7 mg/kg let-7f-5p agomiR, 7mg/kg scrambled miRNA (miR-NC), or 0.2 ml phosphate buffered saline (PBS) on days 1-3 of the 1^st^ , 3^rd^ , 5^th^, and 7^th^ weeks. Firstly, let-7f-5p expression in vertebral bone extracts was measured to verify the effective delivery in mice (**Figure 6A**). We next assessed the characteristics of trabecular bone at the vertebral column. Micro-CT analysis indicated that BV/TV, Tb.N, and Tb.Th in Dex-treated mice were expectedly lower than those in sham-operated control mice, whereas the opposite trend was observed for Tb.Sp. The significant cancellous bone loss was partially reversed after the treatment of let-7f-5p agomiR (**Figure 6B-C**). These putative anabolic effects were verified with the marked effects on bone formation parameters. Histomorphometric measurement showed the markedly upregulation of N.Ob/B.Pm. Meanwhile, the MAR, MS/BS, and BFR/BS were significantly increased in let-7f-5p agomiR mice compared with those in vehicle-treated controls, indicating that bone formation was promotd in let-7f-5p agomiR-treated mice (**Figure 6D**). We also performed ELISA to evaluate the bone turnover. The expression levels of serum markers P1NP and OCN, key markers for bone formation, were markedly increased in let-7f-5p agomiR mice (**Figure 6E**). In addition, injection of let-7f-5p agomiR also prevented estrogen-deficiency-induced osteoporosis and ameliorated age-related bone loss (data not shown). Furthermore, consistent with the results of *in vitro* transfection (**Figure 2**), the expression levels of *Runx2*, *Osx*, *Alp*, and *Ocn* mRNA increased, whereas TGFBR1 expression decreased at both mRNA and protein levels in vertebral samples of let-7f-5p agomiR-treated mice in comparison to those from miR-NC or vehicle-treated mice (**Figure 7**). Taken together, these observations indicate that the *in vivo* therapeutic effect of let-7f-5p is also regulated by TGFBR1 inhibition.

## Discussion

GCs contribute to early and rapid bone loss, and increased fracture risk, thus causing severe socioeconomic problems in societies. Reduced bone formation is a major characteristic of GIOP. For example, low dose GCs rapidly inhibit several key indices of osteoblast activity, such as serum P1NP, propeptide of type I C‑terminal procollagen (P1CP), and OCN 40. The pathophysiology of GIOP is different from that of PMOP, which is characterised by increases in both bone resorption and bone formation, with an imbalance towards bone resorption over bone formation. In the present study, we confirmed that Dex significantly suppressed mouse osteoblast differentiation and mineralisation with significant downregulation of related osteogenic markers (*Runx2*, *Osx*, *Alp*, and *Ocn*), consistent with results of previous studies 15-17. Bisphosphonates (to reduce bone resorption) and teriparatide (to increase bone formation) are currently recommended as first-line therapeutic options for GIOP. In addition, teriparatide is effective at improving bone mineral density compared with oral bisphosphonates and is thus indicated for severe GIOP 1. However, teriparatide, still has some disadvantages, including its relatively high cost, the actual or perceived inconvenience of a daily injection for 24 months, and side-effects that include hypercalcemia, hypercalciuria, and risk of osteosarcom. A number of drugs have also being been suggested to be useful for the regulation of bone formation in GIOP patients, including inhibitors of components of the Wnt signalling pathway (DKK‑1 and sclerostin) 1, 2. However, further elucidation of the mechanisms of osteoblast differentiation under conditions of treatment with GCs, and the development of novel therapeutic targets are still necessary to gain a better understanding of the pathophysiology of GIOP and insight into its prevention and treatment. The results of the present study suggest the GR/let-7f-5p/TGFBR1 regulatory axis may play a critical role in Dex-inhibited osteogenic differentiation of murine BMSCs. In murine models, we also showed that let-7f-5p may be a promising osteoanabolic drug to mitigate pathological bone loss in GIOP patients.

Large numbers of miRNAs regulate protein synthesis by blocking translation of mRNA. Thus, mRNA mediate a wide range of biological processes, including proliferation, differentiation, migration, metabolism, and apoptosis 41. As the first known human family of miRNAs, let-7 and its family members are highly conserved in both sequence and function across species. They also control large numbers of cell-fate determination genes and influence pluripotency, differentiation, tumorigenesis, and transformation 42-45. Further, let-7 family members have prodifferentiation functions with “anti-stemness” properties 46, 47. Indeed, they were reported to promote osteoblast differentiation and repress adipogenesis in human adipose-derived MSCs by directly targeting high-mobility group AT-hook 2 (HMGA2) 20, 48. In addition, let-7 expression was upregulated during osteogenic differentiation of human MSCs 49, 50. The results of our previous study 19 along with those presented here (**Figure 1E**) demonstrated that let-7f-5p expression during osteogenic differentiation in both rat and murine BMSCs, further confirming that let-7 family members are highly conserved among species and play important roles in osteoblast differentiation. Recently, we performed high-throughput sequencing analysis to identify miRNAs involved in GIOP based on sampling human vertebrae, and let-7f-5p was shown to be significantly downregulated, suggesting that let-7f-5p may be a key negative mediator and potential therapeutic target for GIOP. However, the roles of let-7f-5p in rescuing GC-inhibited osteoblast differentiation and bone formation remain elusive. Here, we found let-7f-5p was markedly reduced in the presence of Dex. We further confirmed that let-7f-5p directly targets 3'-UTR of TGFBR1, which was upregulated by Dex treatment. We next focused on the effects of let-7f-5p on the osteogenic differentiation phenotypes of BMSCs. Let-7f-5p in the presence of Dex significantly promoted ALP and mineralisation activity, and upregulated *Runx2*, *Osx*, *Alp*, and *Ocn* mRNA expression.

TGFBR1 is a serine/threonine kinase receptor that transduces downstream signals of members of the TGF-β superfamily 24-26, which plays an important role in bone regeneration 51. For instance, TGF-β promotes osteoclastogenesis 27, 28 and inhibits osteogenesis 29-31. TGF-β signal conduction is dependent on the coordination of heteromeric complexes of TGFBR1 and 2, and specific intracellular Smad effector proteins. Activated heteromeric complexes between R-Smad and Smad4 act as transcription factors and regulate gene transcriptional responses, ultimately inhibiting ALP activity and bone formation. A previous study reported that the TGFBR1 inhibitor SB431542 markedly enhanced osteoblast differentiation of mouse C2C12 cells, and induced the production of ALP and bone sialoprotein as well as matrix mineralisation in human MSCs 32, suggesting that TGFBR1 plays a negative role in bone formation. In the present study, we also confirmed that Dex promoted TGFBR1 expression level in osteogenic differentiation of murine BMSCs. As the specific direct let-7f-5p target, we verified the function of TGFBR1 in let-7f-5p-promoted osteoblast differentiation of Dex-treated cells. Our data showed that TGFBR1 overexpression combined let-7f-5p mimics reversed the promotion of osteoblast differentiation and mineralisation in Dex-treated BMSCs. Taken together, targeting of TGFBR1 by let-7f-5p may be a critical mechanism involved in Dex-inhibited osteogenic differentiation of murine BMSCs.

Most of regulatory roles of GC are coordinated through GR, which was shown to mediate gene expression through several pathways, like interacting as monomeric receptors with DNA-bound transcription factors, such as NF-κB, AP-1, and IRF-3 52. Previously, GC-mediated GR signalling was suggested to play a role in the effects on osteoblasts 2, 53, 54. For example, Sher et al. reported that the expression of osteoblastic GR was necessary for skeletal integrity under physiological status. GC-induced osteoporosis was shown to be regulated by osteoblastic GR, and the monomeric GR could inhibit osteogenesis through reducing the expression of osteoblastic interleukin 11 (IL-11) 54. Conversely, some studies reported that GR-modulating compound, which possessed anti-inflammatory action, has no effect on osteoblast function 55, 56. However, how the interaction between the GR and miRNAs affect osteoblast differentiation in the presence of GCs remains poorly understood. Here, we revealed that GR binded to let-7f-5p promoter and inhibiting the activity. We showed experimentally that there was a marked downregulation of GR-let-7f-5p binding and an accompanying upregulation expression of let-7f-5p in the presence of siGR, indicating that the GR negatively regulates let-7f-5p. GR knockdown studies further confirmed the effective downregulation of TGFBR1 and enhanced development of osteoblast differentiation phenotypes. These findings confirmed that the GR can modulate let-7f-5p when osteogenic differentiation of murine BMSCs is inhibited by Dex.

As let-7f-5p promoted Dex-inhibited osteoblast differentiation *in vitro*, we speculated that it may play an important role in bone formation during GIOP *in vivo*. Here, the potential anabolic action of let-7f-5p was investigated using 6-week-old mice skeletons underwent active modelling. Mice were injected with Dex and let-7f-5p agomiR and miR-NC intermittently more than 8 weeks. Let-7f-5p agomiR protected trabecular bone loss both in GIOP mice and sham-treated controls. Further, bone extracts from mice treated with let-7f-5p agomiR exhibited evidence of increased osteogenic marker (*Runx2*, *Osx*, *Alp*, and *Ocn*) levels and decreased TGFBR1 expression, importantly confirming the *in vitro* findings. These data suggest that let-7f-5p is a potential anabolic target for GIOP treatment. Previous reports yielded conflicting evidence regarding the influence of let-7f-5p on tumour induction and development. Let-7f-5p is reported to promote chemotherapeutic resistance in colorectal cancer 57, was stable or overexpressed in the majority of typical and atypical lung carcinoid samples analysed 58, and contributed to downregulation of thrombospondin-1 in breast cancer with low-dose metronomic paclitaxel chemotherapy 59. Conversely, other groups reported that let-7f reduced the vasculogenic mimicry of human glioma cells by regulating periostin-dependent migration 60; inhibited glioma cell proliferation, migration, and invasion 61; inhibited cisplatin resistance induced by secreted protein acidic and rich in cysteine (SPARC) in medulloblastoma cells 62; was downregulated in hepatocellular carcinoma 63 and papillary thyroid cancer 64; and inhibited tumour invasion and metastasis in human gastric cancer 65. Although no tumours were observed in let-7f-5p agomiR-treated mice in the present study, further preclinical studies of let-7f-5p are required to confirm its safety.

To the best of our knowledge, we demonstrate the roles of let-7f-5p on osteogenesis in BMSCs treated with GCs *in vitro* and on GC-induced osteoporosis *in vivo* for the first time. However, this study had some limitations. Firstly, although we investigated the effect of let-7f-5p in osteogenesis, its role in another important process, osteoclastogenesis, was not examined in the present study. Secondly, the potential mechanism of bone formation is intricate; even though our observations revealed that let-7f-5p targeted TGFBR1 signaling to modulate the Dex-inhibited BMSCs osteogenesis, additional molecules and pathways might also participate in this process. Third, further studies on genetically engineered/large animals are necessary for verifying these findings.

In summary, the results of the present study showed that Dex inhibited let-7f-5p expression and that TGFBR1 is a direct target of let-7f-5p during Dex-inhibited osteogenic differentiation of murine BMSCs. We further showed that let-7f-5p promoted Dex-inhibited osteoblast differentiation *in vitro*. This effect was partly mediated through TGFBR1 targeting, which subsequently mediates the downstream osteogenic transcription. Further, the GR may suppress let-7f-5p expression on the transcriptional level during Dex-inhibited osteoblast differentiation. In addition, let-7f-5p treatment prevented Dex-induced bone loss *in vivo*. The results of this study demonstrate that GR/let-7f-5p/TGFBR1 may be important in GC-inhibited osteoblast differentiation and that let-7f-5p ameliorates GC-induced bone loss, indicating that let-7f-5p would be a potential therapeutic target for GIOP.

## Supplementary Material

Supplementary figure and table.

## Abbreviations

ALP: alkaline phosphatase
α-MEM: α modified essential medium
BFR/BS: bone formation rate
BMSCs: bone marrow-derived mesenchymal stem cells
ChIP: chromatin immunoprecipitation
Dex: dexamethasone
ELISA: enzyme-linked immunosorbent assay
FBS: foetal bovine serum
GCs: glucocorticoids
GIOP: glucocorticoid-induced osteoporosis
GR: glucocorticoid receptor
GRE: GC-responsive elements
HMGA2: high-mobility group AT-hook 2
IL-11: interleukin 11
MAR: mineral apposition rate
miRNAs: microRNAs
MS/BS: mineralizing surface
MT: mutant
negative control: NC
N.Ob/B.Pm: osteoblast number
OCN: osteocalcin
Osterix: Osx
P1NP: procollagen type 1 N-terminal propeptide
P1CP: propeptide of type I C‑terminal procollagen
PMOP: postmenopausal osteoporosis
RT-PCR: reverse transcription PCR
s.d.: standard deviation
siRNA: small interfere RNA
SPARC: secreted protein acidic and rich in cysteine
Tb.N: trabecular number
Tb.Sp: trabecular separation
Tb.Th: trabecular thickness
VOI: volume of interest
WT: wild-type
