# Supplementary material for: Let-7f-5p regulates TGFBR1 in glucocorticoid-inhibited osteoblast differentiation and ameliorates glucocorticoid-induced bone loss
Source: Int J Biol Sci. 2019 Aug 19;15(10):2182–97. doi: 10.7150/ijbs.33490 (PMC6775285; doi:10.7150/ijbs.33490)
Supplement: Supplementary file 1 — Supplementary figure and table. [file ijbsv15p2182s1.pdf]

Supplementary Materials

Table S1

Primers for quantitative RT-PCR.

Gene	Primer sequence
mTGFBR1-F	5’-TCTGCATTGCACTTATGCTGA-3’
mTGFBR1-R	5’-AAAGGGCGATCTAGTGATGGA-3’
mGR (Nr3c1)-F	5’-AGCTCCCCCTGGTAGAGAC-3’
mGR (Nr3c1)-R	5’-GGTGAAGACGCAGAAACCTTG-3’
mRunx2-F	5’- GACCAGTCTTACCCCTCCTA-3’
mRunx2-R	5’-GGCAGTGTCATCATCTGAAA-3’
mOsx-F	5’-AAAGGAGGCACAAAGAAGC-3’
mOsx-R	5’-CAGGAAATGAGTGAGGGAAG-3’
mAlp-F	5’-GCTTTAAACCCAGACACAAG-3’
mAlp-R	5’-AAGAAGAAGCCTTTGAGGTT-3’
mOcn-F	5’-CTCTCTCTGCTCACTCTGCT-3’
mOcn-R	5’-GACTGAGGCTCCAAGGTAG-3’
mβ-actin-F	5’-ACCCAGATCATGTTTGAGAC-3’
mβ-actin-R	5’-GTCAGGATCTTCATGAGGTAGT-3’

Figure S1

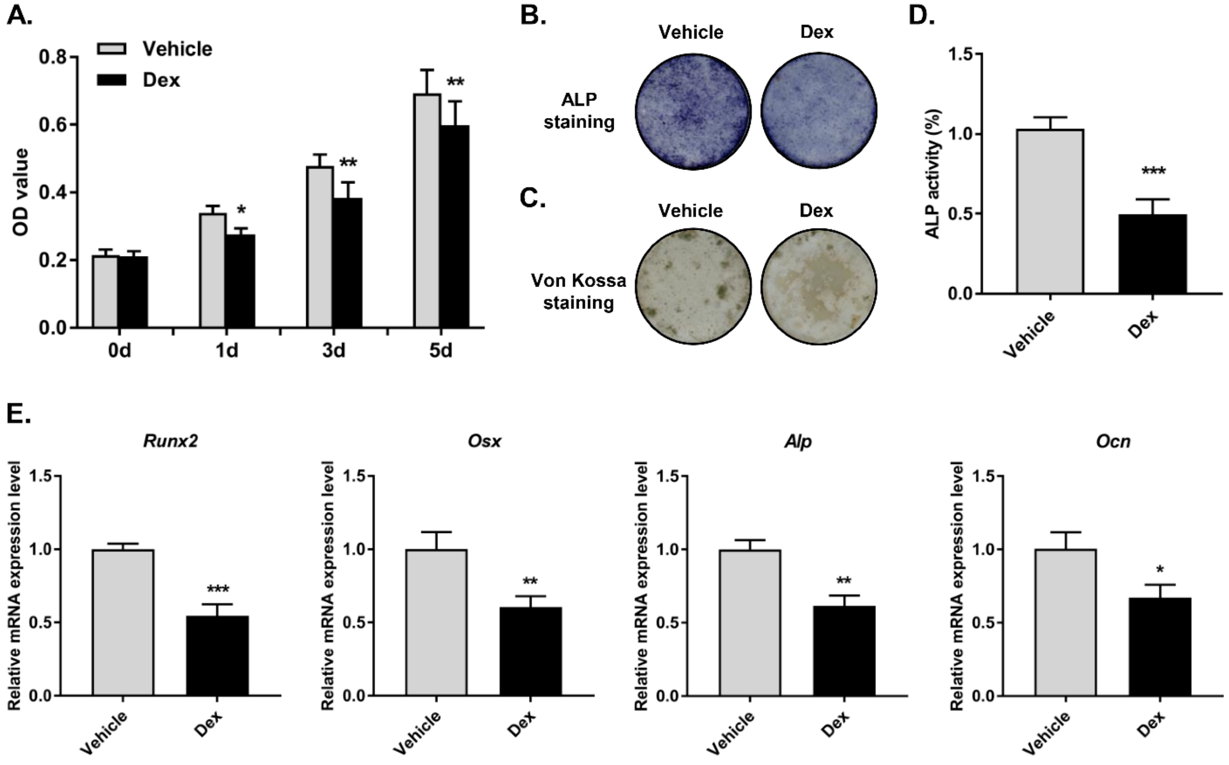

Figure S1. Dex inhibits osteogenic differentiation of BMSCs. (A) Proliferation assay. Dex decreased the proliferation of BMSCs. Cells were cultured in osteogenic induction medium. Results are shown as mean ± s.d. of three independent experiments performed in triplicates, $^{*}P < 0.05$ and $^{**}P < 0.01$ by *t* test. (B-C) Representative images of ALP staining and Von Kossa staining of BMSCs with Dex treatment. (D) Quantitative analysis of the ALP activity in BMSCs with Dex treatment. Results are shown as mean ± s.d. of three independent experiments performed in triplicates, $^{***}P < 0.001$ by *t* test. (E) Quantitative RT-PCR revealed reduced messenger RNA expression of *Runx2*, *Osx*, *Alp*, and *Ocn* in Dex treated BMSCs. Results are shown as mean ± s.d. of three independent experiments performed in triplicates, $^{*}P < 0.05$, $^{**}P < 0.01$, and $^{***}P < 0.001$ by *t* test.
